# Mobile Behavioral Health Coaching as a Preventive Intervention for Occupational Public Health: Retrospective Longitudinal Study

**DOI:** 10.2196/45678

**Published:** 2023-10-20

**Authors:** Sean Han Yang Toh, Sze Chi Lee, Oliver Sündermann

**Affiliations:** 1 Intellect Private Limited Company Singapore Singapore

**Keywords:** mobile health apps, mHealth apps, behavioral health coaching, behavioral coaching, app-based coaching, self-help, employees, well-being, mood, stress, public health, preventive interventions, positive psychology

## Abstract

**Background:**

Researchers have recently proposed that behavioral health coaching (BHC) is effective in promoting proactive care among employees. However, to qualify as a preventive workplace intervention, more research is needed to evaluate whether BHC can further elevate well-being among moderately mentally healthy employees.

**Objective:**

Using real-world data, this study evaluates the preliminary effectiveness of app-based BHC against a nonrandomized control group with open access to self-help tools in improving well-being (ie, mood levels and perceived stress). The study also explores the active ingredients of BHC and dose-response associations between the number of BHC sessions and well-being improvements.

**Methods:**

Employees residing across Asia-Pacific countries (N*=*1025; mean age 30.85, SD 6.97 y) who reported moderately positive mood and medium levels of perceived stress in their first week of using the mental health app Intellect were included in this study. Users who were given access by their organizations to Intellect’s BHC services were assigned to the “Coaching” condition (512/1025, 49.95%; mean age 31.09, SD 6.87 y), whereas other employees remained as “Control” participants (513/1025, 50.05%; mean age 30.61, SD 7.06 y). To evaluate effectiveness, monthly scores from the validated mood and stress sliders were aggregated into a composite well-being score and further examined using repeated-measure conditional growth models. Postcoaching items on “Perceived Usefulness of the BHC session” and “Working Alliance with my Coach” were examined as active ingredients of BHC using 1-1-1 multilevel mediation models. Finally, 2-way repeated-measure mixed ANOVA models were conducted to examine dose-response effects on well-being improvements between groups (coaching and control) across time.

**Results:**

Growth curve analyses revealed significant time by group interaction effects for composite well-being, where “Coaching” users reported significantly greater improvements in well-being than “Control” participants across time (composite well-being: *F*_1,391_=6.12; η_p_^2^=0.02; *P*=.01). Among “Coaching” participants, dependent-sample 2-tailed *t* tests revealed significant improvements in composite well-being from baseline to 11 months (*t*_512_=1.98; Cohen *d*=0.17; *P*=.049). Improvements in “Usefulness of the BHC session” (β=.078, 95% Cl .043-.118; *P*<.001) and “Working Alliance” (β=.070, 95% Cl .037-.107; *P*<.001) fully mediated within-level well-being enhancements over time. Comparing against baseline or first month scores, significant time by group interactions were observed between the second and sixth months, with the largest effect size observed at the fifth month mark (first month vs fifth month: *F*_1,282_=15.0; *P*<.001; η_p_^2^=0.051).

**Conclusions:**

We found preliminary evidence that BHC is an effective preventive workplace intervention. Mobile-based coaching may be a convenient, cost-effective, and scalable means for organizations and governments to boost public mental health.

## Introduction

### Background

Mental health conditions in the workforce are increasingly common and deleterious to employees’ well-being, productivity, presenteeism, compensation claims, and many other occupational outcomes [1-3]. Over the last 2 decades, annual productivity losses caused by these conditions have amounted to approximately US $225 billion for all organizations in the United States [4], £13 billion (US $16.3 billion) in the United Kingdom [5], and US $1.4 to US $1.8 billion across Asia-Pacific countries [6]. To date, most efforts to reduce the burden of these conditions have focused on implementing reactive measures and ensuring immediate treatment for those with manifest difficulties [7] but not proactive care [8]. Not only are these employee assistance programs (EAPs) typically costly and heavily underused (eg, <1% of 10,000 Asian employees attended EAPs [9]; uptake rates are 2.6%-3.5% on average across 133 EAPs in Canada [10]), but cost-effective models suggest that only a third of the overall burden would be alleviated even if treatment were to be delivered successfully to all struggling employees [11]. Consequently, maximizing employees’ well-being through proactive and preventive care may be fundamental to reducing these cost burdens [12].

Recent studies have supported the effectiveness of lower-cost preventive programs in improving well-being across generalized working populations [13]. These interventions can range from fully self-guided mobile interventions [14-19] to personalized behavioral health coaching (BHC) services [20-22]. Contrary to the mixed findings on self-help features [23], BHC interventions have shown consistent improvements in multiple individual-level outcomes. A recent meta-analysis [24] of 18 BHC studies conducted in different samples (ie, students, employees, and the community) revealed significant enhancements in work performance (Hedges *g*=0.19; n=6) and attitudes (Hedges *g*=0.54; n=7), coping self-efficacy (Hedges *g*=0.43; n=10), stress and affectivity (Hedges *g*=0.46; n=10), and self-regulation (Hedges *g*=0.74; n=11). These findings were largely similar to those of a more recent systematic review (n=18) that examined the impact of coaching in purely organizational settings [25]. This review also demonstrated small effect sizes on skill-based outcomes (ie, leadership skills, technical skills, and general competency; Hedges *g*=0.26; n=10), medium effect sizes on well-being (eg, positive emotions, self-efficacy, life satisfaction, and reduction in stress; Hedges *g*=0.46; n=10), and large effect sizes on organizational outcomes (eg, employees’ goal attainment and returns on investments; Hedges *g*=1.15; n=3).

**Figure 1:**
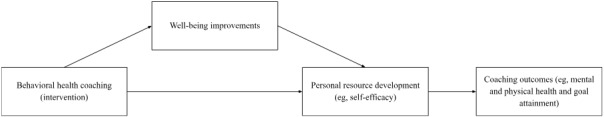
Well-being improvements, a key facet in driving effectiveness of coaching.

By definition, BHC strives to achieve these benefits through a variety of evidence-based approaches including cognitive behavioral therapy (CBT), mindfulness, solution-oriented focus, and positive psychology [26,27]. On the basis of a collaborative, results-oriented relationship, behavioral health care providers are well positioned to address emotional difficulties or life challenges and facilitate positive behavior changes [28,29]. During the course of BHC, well-being enhancements were believed to be a central tenet in building enduring personal resources (physical, intellectual, social, and psychological) [30], which in turn help clients improve their coping abilities and goal striving after each session [31]. The experience of increased positive emotions often leads to the broadening and building of thought-action repertoires. This strengthens metacognition and creativity, which then enables the client or employee to overcome challenges more efficiently and further incites an upward spiral of positive affect and widely improves outcomes [31]. In a similar fashion, decreases in perceptions of stress significantly predicted proactive and preventive coping with future work challenges [32]. Such experiences free up cognitive and self-regulatory resources [33], motivating the client or employee to be further engaged with performance and self-actualization in coaching. Integrating these findings, Figure 1 illustrates these mechanisms.

Despite growing research on the general effectiveness of BHC, potentially important questions related to its preventive effectiveness still remain. Grant et al [34] observed 3 large-scale coaching studies whose participants demonstrated higher-than-average levels of psychopathology [35]. Even though these participants were recruited from nonclinical community samples, at least a quarter of them displayed clinically elevated scores for a variety of psychiatric symptoms [36,37], psychological distress, anxiety, and depressive symptoms at baseline [34]. In supporting the observation by Grant et al [34], most of the studies reviewed by the recent meta-analyses either did not exclude employees at clinically significant risk [38] or did not explicitly state any exclusion criteria [39-41]. Possibly, research is still at an early stage in empirically evaluating BHC as a preventive intervention for positive well-being that tries to sustain or promote mental wellness in healthy individuals at the public health level [42]. This lack of differentiation between psychotherapy and coaching can potentially be detrimental to clients’ well-being as techniques normally executed in BHC (eg, solution-focused strategies and expressive-experiential strategies) may inadvertently cause minimal harm to some at-risk clients. The failure to address clinical levels of stress, anxiety, or depression in BHC may have escalated mental health problems and employee burnout previously [43]. Evaluating the success of BHC as a preventive workplace intervention may then help promote this differentiation. Second, although increments in well-being may catalyze personal resource development during the course of BHC, few studies to our knowledge have examined BHC features that directly cultivate well-being. Working alliance, coach and client personalities, clients’ perceived usefulness of the coach, and clients’ expectancy were coaching-related factors that directly facilitated clients’ resource development and eventually coaching success [44-47]. A plausible question is which of these factors would also cultivate well-being [48,49]. Identifying these predictors may provide insights on how training for behavioral health care providers can be further enhanced or tailored to maximize client outcomes.

Another relatively unexplored dimension is estimating the dose-response relationship between the number of BHC sessions and well-being improvements. The few coaching studies on preclinical employee samples demonstrated significant improvements in various well-being outcomes after 4 to 6 sessions [50,51]. Other studies have revealed similar improvements after 30 to 40 days on average but without knowing the number of sessions [52]. Clearer knowledge on the short- (ie, <4 sessions) and long-term (ie, >6 sessions) effects of BHC can inform expectations and guide treatment decisions for organizations and employees.

### Objectives

The primary objective of this retrospective longitudinal study was to evaluate the preventive effectiveness of a BHC intervention against a no-coaching control group using a sample of employees with at least moderate mental health over a standardized period of 12 months. To our knowledge, this is the first study that tested the effectiveness of app-based BHC on moderately healthy employees. Should BHC participants show significant improvements in well-being overtime, we also explored whether clients’ “perceived usefulness of BHC session” and their “working alliance” with the coach mediated these changes. Finally, we also examined the dose-response relationship between the number of BHC sessions and improvements in well-being, if any.

## Methods

### Study Design

This retrospective study used existing data from pilot commercial collaborations between Intellect and its business partners. Interested employees were enrolled from October 2020, when Intellect’s coaching platform was launched, to October 2022.

### Participants

**Figure 2:**
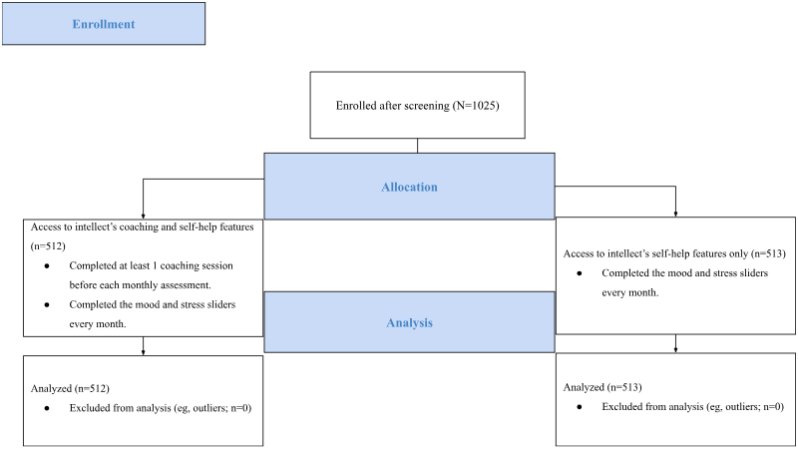
Participant allocation chart.

The Intellect app is publicly available on the Google Play and Apple App stores and has been downloaded >3,500,000 times since its release in October 2019. Interested employees from the commercial pilots voluntarily downloaded Intellect through these app stores after consenting to Intellect’s terms of service and privacy policy. All users were instructed to set up their profiles by completing an onboarding survey regarding their age and gender, the name of their organization, and their personal goals for using Intellect. Although all registered users had access to all self-care features, only a subsample of them gained additional access to BHC services depending on their organization. Registered users were further screened for study eligibility, which included (1) being aged ≥18 years and (2) having scored an average daily rating of “≥2” on the mood slider and “≤3” on the stress slider (see the following section) in their first week of use. To compute monthly changes in well-being, eligible participants were also expected to (3) attempt the well-being scales every 4 weeks. Users in the “Coaching” condition would (4) have participated in at least one BHC session every month, whereas the remaining users in the “Control” condition may or may not have engaged with Intellect’s self-care features during the month. All “Coaching” users were prompted to complete a 4-item postcoaching feedback form after each BHC session. In accordance with Intellect’s risk assessment procedures, it may be reasonable to assume that these “Coaching” users were “mentally healthy.” As a qualifying criterion, each behavioral health coach would have been trained to detect symptoms of anxiety and depression and accumulated a minimum of 300 client hours before they were employed at Intellect. Users who were identified as showing elevated symptoms of anxiety and depression in the first session based on each provider’s professional judgment would be referred out of the BHC program to see an internal (in-app) or external licensed therapist. These users were not considered as having completed 1 coaching session. Therefore, they are not present in this retrospective cohort. The overall participant flowchart is shown in Figure 2. The resulting sample of 1025 participants comprised predominantly women with a mean age of 30.85 (SD 6.97; range 18-61) years. To maintain the real-world evaluation of this data set, participants received neither any in-app reminders to engage with the app nor monetary reimbursement for their participation.

### Intervention

#### Self-Guided Features

**Figure 3:**
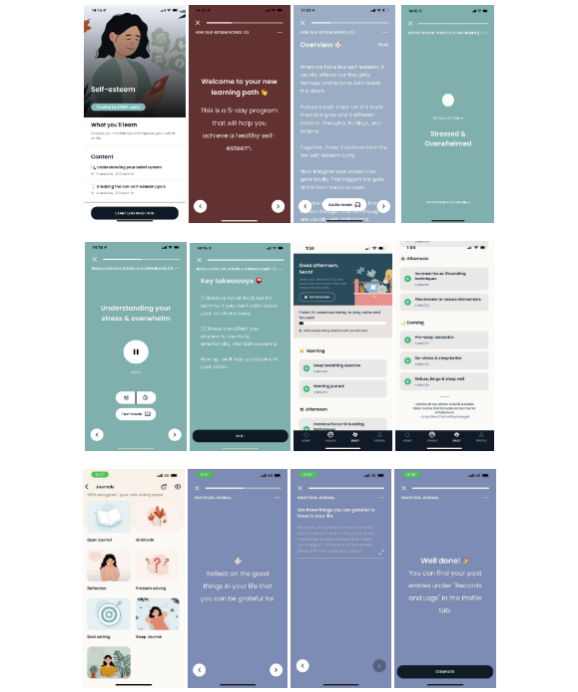
Intellect’s self-help exercises (learning paths, rescue sessions, toolkits, and journaling).

Intellect is a consumer-based mental health app that provides all registered users with free access to a variety of self-guided features, some of which have been validated in previous randomized controlled trials [18,19,53,54]. On the “Home” tab, participants can access any of the 3 self-help features: “Learning Paths,” “Rescue Sessions,” or “Guided Journals” (Figure 3). “Learning Paths” contains psychoeducation on different aspects of mental health. An example is the “Self-Esteem Learning Path,” where the program guides users to identify and cognitively restructure their negative thoughts related to self-perceptions. A “Rescue Session” targets a specific theme of adversity, such as “procrastination,” “irregular sleep,” “burnout,” or “loneliness.” Depending on the adversity, the theoretical foundations of these sessions can range from mindfulness and self-compassion practices to CBT and active behavior change techniques (eg, self-monitoring and social support). Intellect’s “Guided Journals” comprise 6 themes: “gratitude,” “reflection,” “problem-solving,” “goal-setting,” “sleep,” and “self-affirmation.” Each journal provides specific guidance to each participant on how to write a relevant entry. For example, the “gratitude” journal encourages each participant to recall something that they can be thankful for on that day but not usually on other days. The fourth feature, “Toolkit,” may be most suitable for participants who prefer a standardized schedule for self-care. This feature, which is accessible on the “Daily” tab, contains brief mental and physical exercises dedicated to 3 parts of the day (Figure 3). The morning section includes deep breathing exercises guided by therapist-led audio messages. The afternoon section provides grounding techniques that increase mindful focus on the users’ surroundings. Finally, the evening section focuses on improving sleep through meditation and cognitive defusion.

#### BHC Component

**Figure 4:**
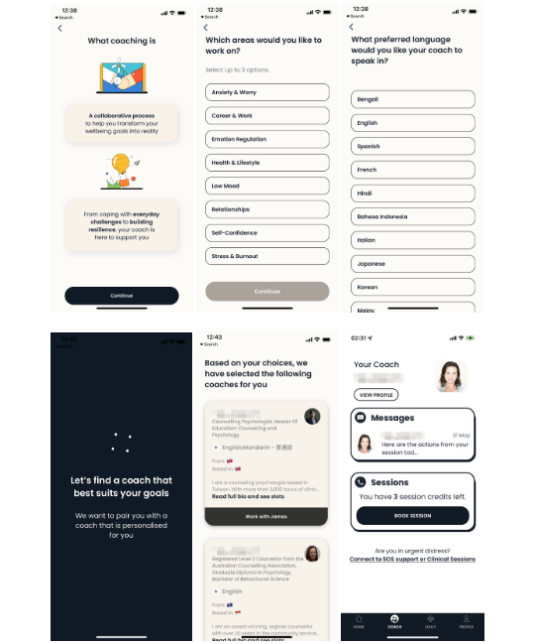
Screenshots of Intellect’s behavioral health coaching, accessible on the Coach tab.

Intellect also offers personalized one-on-one app-based coaching to support each participant’s personal and professional development. Behavioral health care providers across the Asia-Pacific region are connected to the platform, where each of them receives professional certification either in counseling from registered institutions or in coaching licensed by the International Coaching Federation [55]. The former group constitutes approximately 85% of all Intellect’s providers. Specifically, these coaches are accredited at a minimum with a master’s degree in counseling and are officially licensed by their national counseling entities (eg, the Singapore Association for Counselling) to practice professionally. A minimum of 300 client hours are also required to qualify as a behavioral health coach for Intellect. Intellect’s diverse coaching network means that providers have different specialties. Most of Intellect’s providers are trained in CBT or problem-solving strategies and third-wave CBT approaches such as mindfulness, dialectical behavior therapy, or acceptance and commitment therapies. Participants supplied with “coaching” credits by their organizations gain access to BHC services via the “Coach” tab. Upon accessing the tab, users are automatically directed to select 3 areas of well-being that they may be interested to work on with a coach (ie, “anxiety & worry,” “career & work,” “emotional regulation,” “low mood,” “health and lifestyle,” “relationships,” “self-confidence,” and “stress & burnout”) and their preferred language of communication. Coach selection is then optimized through a set of algorithms that make recommendations based on the participants’ motivations, goals, app engagement patterns, preferred language, and coaching style. Each coaching session, which lasts approximately 30 minutes, is conducted via video between the coach and the user on the same tab. Typically, in the first session, the coach attempts to fully understand each user’s topical struggles and formulate clear goals for the coaching journey. For subsequent sessions, the coach is free to explore and select the evidence-based modalities and exercises that they think would work best to fulfill the user’s set goals and presenting struggles. Hence, the delivery of Intellect’s BHC program is flexible and not in a standardized modular format. Between each session, the coach and user may continue to interact via SMS text message and audio messaging, usually to clarify homework or provide additional motivational support. Coaching sessions are typically arranged a minimum of a week apart to allow for more time for implementing coaching exercises and reflection. These features are illustrated in Figure 4.

The collaborative and supportive relationship between the coach and client is thought to be critical in attaining valued outcomes [56]; hence, all providers were trained in areas of active listening, goal setting, empowerment, scaffolding behavior changes, providing constructive feedback, emotional validation, and maintaining accountability of change. During a typical session, the user may be encouraged to lead discussion topics related to the mutually agreed upon goals as this increases responsibility over their personal development at a comfortable pace. In response, the coach takes a future-focused stance when facilitating the discussion, empowering the user to discover potentially novel solutions. As a form of preventive care, BHC actively seeks to develop growth mindsets toward positive behavior change. Once this is accomplished within the session, both the coach and user proceed to formulate a feasible action plan beyond the session. The coach continues to provide support in monitoring progress and reframing unhelpful thinking styles while emphasizing accountability of change for the user.

#### Measures

##### Overview

Participants rated their emotions and perceived stress levels using a mood and stress slider, respectively. These sliders were located at the top of the “Home” tab. Participants were able to redo their ratings at any period of the day, and these scores were refreshed on a daily basis.

##### Perceived Stress

The self-developed stress slider was rated on a 5-point scale (1=“Very Low,” 2=“Low,” 3=“Medium,” 4=“High,” and 5=“Very High”). Previously, we examined the convergent validity of this self-developed item using a separate large sample of working professionals (N=997) recruited from the web-based Rakuten platform in an unpublished study. The Rakuten platform functions similarly to the Amazon Mechanical Turk platform in the United States. We found significant positive correlations (*r*=0.390; *P*<.001) and acceptable factor loadings (λ=0.68) between the stress slider and Perceived Stress Scale items [57]. On this scale, we considered scores of ≥4 to be “unhealthy” and scores of ≤3 to be “healthy.” This is consistent with our screening criterion.

##### Mood Levels

Similar to the stress slider, the self-developed mood slider was rated on a 5-point scale (0=“Terrible,” 1=“Very Bad,” 2=“Alright,” 3=“Good,” and 4=“Fantastic”). Higher total scores indicate a more positive mood. Previously, significant negative correlations between the mood slider and Patient Health Questionnaire–4 items (*r*=−0.501; *P*<.001) and high factor loadings (λ=0.72) indicated good support for convergent validity [58]. On this scale, we considered scores of ≥2 to be “healthy” and scores of ≤1 to be “unhealthy.” This is also consistent with our screening criterion.

##### Composite Well-Being Score

As the use of single-item measures may be less reliable in assessing a multidimensional construct such as well-being [59], participants’ scores on the mood and stress sliders were aggregated to form a composite well-being score. All stress scores were reverse coded such that higher aggregate scores on composite well-being indicated greater mental health (ie, better mood and lower stress). In line with the scoring systems of the 2 items, we considered scores of ≥5 (ie, 3 on stress and 2 on mood) to be “healthy” and scores of ≤4 to be “unhealthy.”

##### Postcoaching Feedback

To evaluate acceptability, “Coaching” users were prompted to complete a self-developed 4-item feedback form immediately after a coaching session. A total of 2 items on “Perceived Usefulness of Coaching” asked whether participants (1) found the session helpful and (2) felt closer to their personal goals after the session. The remaining 2 items on “Perceptions of Working Alliance” asked whether (1) participants felt supported and understood by their respective coaches and (2) the coach generally helped them gain new insights and skills. Each item is rated on a 5-point scale (1=“Strongly Disagree,” 3=“Neutral,” and 5=“Strongly Agree”). Among the coaching users, this form showed excellent internal consistency (Cronbach α=.81).

### Statistical Analyses

Descriptive statistics, including the average retention period for each group, were calculated using SPSS Statistics (version 28.0; IBM Corp) [60]. For intervention effects on primary outcomes, a mixed model for repeated-measure conditional growth model analysis was conducted to test whether differences in the change trajectories on well-being between participants could be explained by group membership (condition). Analyses were conducted using the *lme4* package on R (R Foundation for Statistical Computing) [61]. The use of multilevel mixed models allows for not only missing data but also time-varying covariates [62]. For samples with high attrition, multilevel models can be approximated using maximum likelihood estimators. These estimators provide unbiased parameter estimates as long as the missing or dropout data are random [63-66]. As the intervention was conducted in observational (nonexperimental) settings, it is expected for attrition rates to be very high in this sample. Hence, the use of multilevel models may be a reliable method to estimate the true intervention effect of Intellect’s BHC services against a “no-coaching” control group over the period of evaluation (ie, 12 mo) [67]. In the mixed model, group, time, time by group interaction, and covariates (variables presenting significant differences between the “Coaching” and “Control” groups at baseline, demographic characteristics, and number of times each self-guided feature [ie, learning paths, rescue sessions, journaling sessions, and toolkits] was engaged with/mo) were fitted as fixed effects in the model. Participants were included as random intercepts. The “Group” variable was coded binarily (0=“Control”; 1=“Coaching”), whereas “Time” indicates the number of months since participants started using the app. Before these variables were included in the model or models, mean centering was necessary to compute a more accurate cross-level interaction by separating within- from between-subject effects [68,69]. “Group,” “Time,” and demographic variables (age and gender) were all grand mean centered to provide meaning to their intercepts. The grand mean centering of “Group” and “Time” also reduces multicollinearity with their interaction term through decorrelating predictor variables [70]. As purely within-subject variables, frequencies of engaging with each self-guided feature were group mean centered.

**Figure 5:**
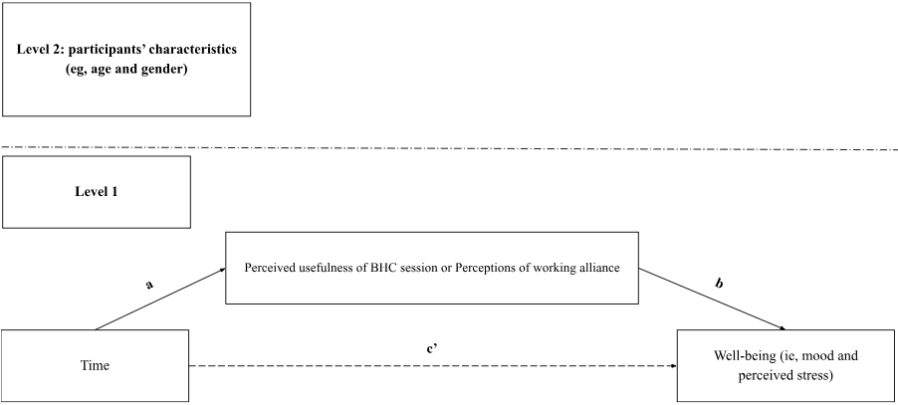
Illustration of the 1-1-1 mediation model. Path a represents the effect of time on clients’ perceptions of behavioral health coaching (BHC). Path b represents the changes in well-being when client ratings of BHC change and time is held constant. Path c′ represents the direct effect of time on well-being when controlling for the effect of changes in clients’ perceptions. Path a*b represents the within-group indirect effect of time on well-being represented by changes in the clients’ perceptions of BHC.

To examine whether changes in “perceptions of coach” and “perceived usefulness of the BHC session” mediated the relationship between time and primary outcomes for the “Coaching” users, 1-1-1 multilevel mediation models [71] were estimated using the *MLmed* function in SPSS [72]. Similar to linear mixed models, multilevel mediation allows all participants to be included regardless of missing data. As mentioned previously, the restricted maximum likelihood estimators of these models also allow for the approximation of unbiased parameter estimates in the presence of high attrition. Essentially, multilevel mediation models have the advantage to exploit all valid data points [73]. They also extend classic mediation models to account for the nested nature of longitudinal data (ie, time nested within individuals). Mediators were group mean centered to evaluate within-subject effects, whereas parameters were estimated using restricted maximum likelihood estimation. Each model also included the same covariates as in the linear mixed models. Figure 5 illustrates the 1-1-1 multilevel mediation model.

Finally, to test for well-being improvements between specific time points, 2 × 2 repeated-measure mixed ANOVA models with time (first month [T1] and second, third, fourth, fifth, sixth, seventh, eighth, ninth, 10th, 11th, or 12th month [T2]) as the within-subject factor and group (coaching and control) as the between-subject factor were conducted to assess the difference in participants’ well-being scores. In each ANOVA model, demographic variables (age and gender), quantity, and type of self-guided feature engagement were controlled as covariates. A significant time by group interaction effect indicates group differences in improvement over the 2 time points. Any significant interaction effects were further examined using Bonferroni-adjusted contrast analyses to assess the locus of differences for each group across the time points.

### Ethical Considerations

This study received an advisory review from the Advarra Institutional Review Board and was deemed to be of no more than minimal risk (protocol Pro00069682). Employees downloaded the app after having agreed to Intellect’s terms of service and privacy policy, which included consent to use anonymized data for research purposes. All retrospective cohort data were deidentified.

## Results

### Participants

Of the total 1025 users, participants were predominantly woman (n=626, 61.07%; mean age 30.85, SD 6.97 y) who completed close to 5 monthly assessments on average (mean 4.92, SD 6.07). At baseline, the average participant experienced moderate levels of perceived stress (mean 2.39, SD 0.762) and mood (mean 2.98, SD 0.966). Within the control group, 71.3% (366/513) of the participants scored moderately for mood, whereas the remaining 27.1% (139/513) and 1.6% (8/513) experienced “Good” and “Fantastic” mood at baseline, respectively. Similarly, 41.5% (213/513) of the control participants experienced moderate levels of stress, whereas the remaining 47.4% (243/513) and 11.1% (57/513) scored “Low” and “Very Low” for baseline stress levels, respectively. Within the intervention group, 68.8% (352/512) of the participants experienced moderate mood levels, whereas the remaining 25% (128/512) and 6.3% (32/512) scored “Good” and “Fantastic” for mood levels, respectively. For stress levels, 58.4% (299/512) of the intervention participants scored “Moderate,” whereas 35.5% (182/512) and 6.1% (31/512) scored “Low” and “Very Low” at baseline, respectively. Table 1 presents the main descriptive statistics and baseline scores of the groups. Apart from the lower number of monthly assessments completed on average by “Control” participants (mean difference −1.76, SD 0.180; *t*_4662_=−9.82; *P*<.001), independent-sample 2-tailed *t* tests and chi-square analyses did not reveal significant differences in other baseline characteristics (see Table 1). Independent-sample 2-tailed *t* tests and chi-square analyses also revealed no significant differences in any demographic (age and gender) measures between participants who retained their engagement with Intellect and those who withdrew from the app over the 12 months (*P*>.05 in all cases). This result also suggests that missing data were random and the use of restricted maximum likelihood estimators in our multilevel models was adequate [66].

**Table 1:** Baseline descriptive statistics for demographics (N=1025).

Variable		Coaching (n=512)	Control (n=513)	*P* value	
Age (y), mean (SD)		31.09 (6.87)	30.61 (7.06)	.27	
Monthly assessments completed, mean (SD)		5.62 (6.47)	3.86 (5.24)	<.001	
**Well-being (averaged across the first week), mean (SD)**					
	Perceived stress	2.39 (0.729)	2.39 (0.792)	.92	
	Positive mood	2.94 (1.07)	3.02 (0.852)	.48	
	Composite well-being	5.64 (0.151)	5.43 (0.164)	.35	
**Gender, n (%)**					.16
	Woman	326 (63.7)	300 (58.5)		
	Man	170 (33.2)	194 (37.8)		
	Nonbinary	7 (1.4)	14 (2.7)		
	Other	4 (0.8)	5 (1)		

### Engagement With Self-Guided Features

As shown in Table 2, there were no significant differences in the average number of self-guided features engaged with per month between the 2 groups (*P*>.05 in all cases). Relative to those in the “Control” group (mean 5.18, SD 21.5), “Coaching” participants engaged with and completed an average of 5.06 (SD 9.54) self-guided features per month. Similarly, there were almost no significant differences in the average number of features engaged with per month between the 2 groups except for the first month (learning paths: mean difference −0.098, SD 0.046, *t*_1022_=−2.13; *P*=.03; journaling sessions: mean difference −0.293, SD 0.118, *t*_1022_=−2.48; *P*=.01; rescue sessions: mean difference −0.502, SD 0.132, *t*_1022_=−3.81; *P*<.001). Only at baseline were “Coaching” participants significantly more engaged with nearly all self-guided features (learning paths: mean 0.82, SD 0.755; journaling sessions: mean 0.88, SD 2.04; rescue sessions: mean 1.08, SD 2.63) than “Control” participants (learning paths: mean 0.72, SD 0.714; journaling sessions: mean 0.59, SD 1.73; rescue sessions: mean 0.58, SD 1.40). The descriptive results of each self-guided feature can be found in Tables S1-S4 in Multimedia Appendix 1.

**Table 2:** Monthly average number of self-guided features engaged with between the groups.

Period by month	Coaching (n=512), mean (SD)	Control (n=513), mean (SD)	*P* value
First month (N=1025, 100%)	4.59 (6.87)	3.79 (11.7)	.19
Second month (N=1025, 100%)	3.94 (7.99)	3.08 (11.7)	.17
Third month (n=709, 69.17%)	3.60 (8.40)	3.89 (15.3)	.75
Fourth month (n=456, 44.49%)	4.08 (8.57)	5.91 (20.3)	.18
Fifth month (n=301, 29.37%)	4.89 (9.92)	7.51 (26.2)	.21
Sixth month (n=218, 21.27%)	5.27 (10.3)	7.25 (30.5)	.48
Seventh month (n=156, 15.22%)	6.79 (12.0)	11.5 (37.3)	.23
Eighth month (n=123, 12%)	6.61 (11.8)	12.5 (42.1)	.23
Ninth month (n=92, 8.98%)	8.37 (17.3)	16.8 (52.5)	.25
10th month (n=75, 7.32%)	7.31 (11.2)	20.9 (57.9)	.09
11th month (n=60, 5.85%)	7.50 (10.1)	21.5 (59.8)	.14
12th month (n=53, 5.2%)	7.63 (11.8)	26.9 (79.9)	.14

### Effects of BHC on Well-Being

#### Overview

**Figure 6:**
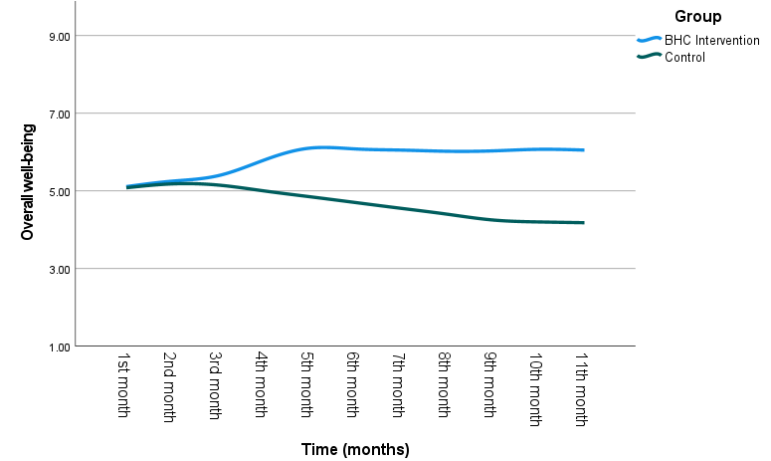
Changes in well-being over time (mo) as a function of group. BHC: behavioral health coaching.

The fixed effects of time, group, time × group interactions, and covariates (ie, self-guided features) are presented in Table 3. Significant effects of time × group interaction were found for composite well-being, indicating that change patterns for well-being scores differed significantly between the “Coaching” and “Control” participants across time (well-being: *F*_1,391_=6.12; η_p_^2^=0.02; *P*=.01). Significant main effects were also found for group (well-being: *F*_1,354_=7.40; η_p_^2^=0.02; *P*=.007), with the “Coaching” group generally exhibiting greater well-being than the “Control” group on average over the 12 months. There was no significant main effect of time on well-being (*F*_1,391_=2.35; *P*=.13). Simple slope analyses were conducted on SPSS to probe the interaction effect, namely at high (1 SD above the mean at 11 mo) and low (1 SD below the mean at baseline) levels of “Time.” Figure 6 shows the best-fit lines indicating changes in well-being over the months as a function of group. Probing revealed a significant improvement in composite well-being from baseline to 11 months for the “Coaching” group (*t*_512_=1.98; Cohen *d*=0.17; *P*=.049), whereas no significant changes were observed for “Control” participants over the same period (*t*_513_=−1.50; Cohen *d*=−0.13; *P*=.14).

**Table 3:** Fixed effects on well-being.

Primary outcome and effect		β (SE; 95% Cl)	Partial η^2^	*P* value
**Well-being**				
	Time	−.001 (.014; −.012 to .010)	0.00597	.13
	Group	.362 (.103; .239 to .485)	0.02	.007
	Time × group	.067 (.027; .047 to .088)	0.02	.049
	Learning paths	.005 (.071; −.075 to .077)	0.0000409	.55
	Rescue sessions	−.076 (.027; −.106 to –.052)	0.02	.005
	Journaling	.023 (.012; −.001 to .048)	0.00436	.06
	Toolkits	.000 (.010; −.010 to .010)	0.00000256	.98
	Age	.015 (.006; .008 to .023)	0.02	.01
	Gender	−.019 (.082; −.126 to .88)	0.000113	.84

#### Multilevel Mediation Models and Coaching Service Satisfaction

Table 4 presents the path coefficients and the direct and indirect effects of the postcoaching feedback items on well-being scores. Among the large subsample of “Coaching” users who completed the postcoaching feedback items (341/512, 66.6%; mean age 31.52, SD 7.21 y), these participants developed more positive perceptions of their coach (ie, “Perceptions of My Coach”) and found the BHC sessions to be increasingly useful (ie, “Perceived Usefulness of Coaching”) over time. Increases in both “Perceived Usefulness of Coaching” (well-being: β=.078, 95% Cl .043-.118; *P*<.001) and “Working Alliance” (well-being: β=.070, 95% Cl .037-.107; *P*<.001) fully mediated the within-level improvements in well-being during the intervention period (Table 4). Across the sessions, 88.3% (301/341) and 84.2% (287/341) of these participants averaged a high score of ≥8 out of 10 on “Perceived Usefulness of Coaching” and “Perceptions of Working Alliance,” respectively, implying a high level of satisfaction with the BHC intervention. The high average ratings on “Perceived Usefulness of Coaching” were supported by high scores on its individual items. On “Helpfulness” of coaching, 0.6% (2/341), 5% (17/341), 15% (51/341), and 79.5% (271/341) of these participants averaged a rating of 1, ≤3, ≤4, and ≤5 across the monthly sessions, respectively. On “Goal Attainment,” 0.3% (1/341), 1.2% (4/341), 12.9% (44/341), 26.1% (89/341), and 59.5% (203/341) of these participants averaged a rating of 1, ≤2, ≤3, ≤4, and ≤5 across the monthly sessions, respectively. Similarly, high monthly average ratings for “Perceptions of Working Alliance” were supported by high scores on its items. On “Supportiveness of my Coach,” 0.3% (1/341), 2.6% (9/341), 17.3% (59/341), and 79.7% (272/341) of these participants averaged a monthly rating of 1, ≤3, ≤4, and ≤5, respectively. Finally, on “Initiative of my Coach,” 0.6% (2/341), 0.6% (2/341), 8.2% (28/341), 25.2% (86/341), and 65.4% (223/341) of these participants averaged a monthly rating of 1, ≤2, ≤3, ≤4, and ≤5, respectively.

**Table 4:** Multilevel coefficients and indirect effects of postcoaching feedback items on well-being.

Mediator	Path a^a^	Path b^b^	Path c’^c^	Indirect effects
				Path “ab”^d^ (95% Cl)
Perceived usefulness of coaching	0.118^e^	0.664^e^	−0.062	0.078^e^ (0.043-0.118)
Perceptions of working alliance	0.105^e^	0.671^e^	−0.057	0.070^e^ (0.037-0.107)

^a^The effect of time on the mediator (*Perceived Usefulness of Coaching or Perceptions of Working Alliance*).

^b^The effect of the mediator on well-being.

^c^The direct effect of time on well-being after controlling for the indirect effects of the mediator.

^d^The indirect effects of the mediator on the relationship between time and well-being.

^e^*P*<.001.

### Mixed ANOVAs (Exploratory Analyses)

Significant time by group interactions were observed between the second and sixth months, contrasting with participants’ well-being scores at baseline or first month, with the largest effect size observed at the fifth month mark (first month vs second month: *F*_1,1004_=10.04; *P*=.001; η_p_^2^=0.010; first month vs third month: *F*_1,690_=9.23; *P*=.002; η_p_^2^=0.013; first month vs fourth month: *F*_1,436_=11.3; *P*<.001; η_p_^2^=0.028; first month vs fifth month: *F*_1, 282_=15.0; *P*<.001; η_p_^2^=0.051; first month vs sixth month: *F*_1,200_=7.01; *P*=.009; η_p_^2^=0.034). There were no significant time by group interactions between the seventh to 12th month scores and baseline scores across both groups (*P*>.05 in all cases). Results from post hoc pairwise comparisons revealed significant increases in well-being from the first month leading to the sixth month in the “Coaching” group (first month vs second month: mean difference 0.231, SD difference 0.043; *P*<.001; first month vs third month: mean difference 0.666, SD difference 0.102; *P*<.001; first month vs fourth month: mean difference 0.237, SD difference 0.081; *P*<.001; first month vs fifth month: mean difference 0.420, SD difference 0.069; *P*<.001; first month vs sixth month: mean difference 0.816, SD difference 0.204; *P*<.001). In contrast, the “Control” participants did not experience any significant well-being improvements across any time points (*P*>.05 in all cases).

## Discussion

### Principal Findings

This study demonstrated the preliminary effectiveness of a retrospective BHC intervention in improving the well-being of relatively mentally healthy employees. Consistent with the few face-to-face BHC interventions that manifested several well-being benefits (ie, self-reported physical health, burnout, psychological distress, and positive and negative affect) in employees with minimal mental health problems at baseline [74,75], our findings also showed that web-based, one-on-one BHC may replicate some of these benefits. As face-to-face programs incur higher costs than web-based interventions, which, in turn, limits their scalability and feasibility in a large-scale rollout [76], mobile-based or web-based coaching sessions may be a more convenient, cost-effective, and scalable means for organizations and governments to promote public mental health. In line with previous research [77], our results also showed that having free access to self-help tools may not necessarily be sufficient to sustain mental wellness among healthy employees, as indicated by our finding that the well-being levels of noncoaching users declined over time (Figure 6). Previously, the role of human contact and therapeutic alliance with a professionally trained coach has improved participants’ adherence rates to the intervention [78,79]. This is also supported by the higher number of monthly assessments completed by the average “Coaching” participant in this study. Compared with coaching, self-help features were less able to be contextualized to the needs of every individual user, which may then lead to lower engagement motivation with the app [79,80]. Given that mentally healthy individuals are already prone to perceive a lower need for support and intervention [81], self-guided features that are often heavily reliant on users’ self-awareness and motivation at baseline may be limited in their capacity to challenge growth and cultivate health and well-being–promoting behaviors and thoughts. Comparatively, most behavioral health coaches are professionally trained and committed to increasing clients’ introspection on their needs and goals after accounting for their background factors (eg, specific vulnerabilities, working habits, communication styles, and content of stressors) [46]. Even for flourishing clients with minimal needs, behavioral health coaches could leverage their character strengths and encourage positive habit formation to maintain wellness [82,83]. Although it is beyond the scope of this study, future comparative research may consider delineating the mechanisms that derive higher preventive effectiveness in BHC as compared with self-help interventions. Finally, owing to the nonsignificant between-group differences in the monthly engagement rates of self-guided features, we infer that these positive effects were more likely to be derived from the coaching sessions.

Among the “Coaching” participants, our study also demonstrated that clients’ perceptions on the “usefulness of BHC sessions” and “working alliance” significantly mediated their improvements in well-being over the course of BHC. First, our findings that these perceptions were developed across time (“a” path; Table 4) are consistent with the notion that coaching is a reflective, gradual process that transforms behavior by cultivating the client’s self-awareness of a need to thrive. Accordingly, a stable “working alliance” involves an ongoing, mutual exchange of sincerity, respect, and trust between the coach and the client [84-87]. Even for coaches who showed genuine interest in the client’s well-being and goals from the first session, establishing clear agreements and maintaining accountability of changes in later sessions were necessary components of a client-rated effective alliance [88,89]. These positive developments in alliance, when combined with effective coaching techniques, may incrementally contribute to the higher client-perceived usefulness of coaching sessions as they progress toward attaining their goals [90,91]. Our findings that these processes fully mediated improvements in well-being, which in turn propels other “active ingredients” of BHC, highlight the importance of fostering these components in current BHC practices. Future work may continue to explore other mediators of change in well-being and suggest enhancements to BHC training.

In line with recent web-based coaching studies [50,51], our study revealed larger effect sizes for employees who completed 4 to 6 BHC sessions. We extend these findings by also showing that significant increments in well-being were possible after only 2 to 3 sessions. These numbers differed from the recommended range of CBT sessions for patients with mental health diagnoses (ie, 8-20 sessions [92]), presuming that healthy functioning individuals may find it relatively easier to adhere to the intervention. Imminent improvements after the initial sessions can be attributed to a variety of reasons, such as establishing a trustable working alliance [93], enhanced self-awareness, and relief of the user’s own presenting struggles during problem construction and goal formulation [94]. Gaining clarity and resolution of their problems subsequently enhances motivation, interest, and hope for intervention success in the client. This, in turn, strengthens participant adherence in later sessions, which is helpful in maximizing outcomes [95]. However, the larger effect sizes observed for this treatment length may also be due to higher levels of motivation or different presenting problems that may not require more sessions to reach a resolution. These potential confounding variables were not assessed in our study. For the same reasons, the absence of significant improvements starting from the seventh session may not necessarily imply a lack of long-term effects of a BHC intervention. As this was a retrospective cohort study, we recommend that future researchers use more rigorous research designs, such as a 2-armed randomized controlled trial collecting objective well-being data from wearable devices to assess the dose-response relationship.

### Strengths and Limitations

Despite the large sample size and statistically rigorous methodologies, this study also has several limitations. First, the lack of randomization procedures precluded any possible conclusions on the causality of the intervention. Second, although this study used real-world data that not only enhanced external validity but also allowed for an appropriate assessment of the acceptability and preliminary impact of Intellect’s coaching, users were not required to complete some assessments (ie, postcoaching feedback), which limited the evaluation of possible mediators to a small subsample. Third, participants may have used other well-being apps in conjunction with Intellect’s BHC or self-guided features during the study, which may have confounded our findings. Fourth, although our self-developed items were validated using an external sample (n=997), existing surveys that were validated thoroughly and cross-culturally (ie, the Patient Health Questionnaire–2 and Perceived Stress Scale) may have increased the reliability of our findings. Fifth, the use of single-item scores on mood and stress as one of the screening criteria for “mentally healthy” employees is much less reliable in comparison with more robust screening procedures using clinically validated scales (eg, the Patient Health Questionnaire–9). Even if participants in the intervention group had been subjected to some form of risk assessment by their providers, no formal assessments were conducted in this study. This may have affected the homogeneity of the groups, which would bias the group comparison. Sixth, despite having already controlled for self-guided feature engagement as a covariate in our main analyses, the average improvements in well-being could still be influenced by possible web-based effects between the BHC intervention and self-guided features. These limitations, together with the small effect sizes, warrant the consideration of our results as only preliminary. Future research aiming to replicate our findings may benefit from a more rigorous design, such as including randomized treatment and control groups and assessing all participants using brief psychometrically validated instruments at standardized time points. For instance, with a 3-armed randomized controlled design, researchers have a better chance of parsing the main effects of the BHC intervention, self-guided feature engagement, and no treatment on well-being outcomes separately. Unlike real-world studies, a controlled research study can also prevent its participants from receiving alternative interventions (ie, self-guided features and other well-being apps) in parallel and minimize confounding effects. Finally, this study has limited external validity to the working population at large as the sample consisted mainly of relatively young (mean age 30.85, SD 6.97 y) woman employees (626/1025, 61.1%) residing across Asia-Pacific countries. The transferability of our findings can be further strengthened if researchers can replicate our study with more diverse samples (eg, European or Western employees, Asian male employees, and older employees).

### Conclusions

Using a large sample of relatively mentally healthy employees residing across the Asia-Pacific region, this study evaluated the real-world effectiveness of Intellect’s professional coaching services against a nonrandomized control group with open access to Intellect’s self-help features in further improving well-being. The findings indicated significant improvements in composite well-being among healthy employees who participated in coaching, and no significant between-group differences were found in the monthly engagement rates of self-help features. Within a subsample of the intervention group, clients’ positive perceptions of “working alliance” and “usefulness of coaching” mediated the improvements in well-being. Our findings are useful given the potential for scaling up easily accessible, cost-effective coaching services to many organizations that currently aim to promote preventive and proactive care for their employees. Given the lack of randomized groups, comprehensive psychometric instruments, a more diverse sample, and other possible confounds, this study provides only preliminary evidence that BHC can improve occupational welfare, which translates to significant improvements in public mental health.

## Appendix

Multimedia Appendix 1 Tables comparing the differences in engagement with each self-guided feature (learning paths, journaling sessions, rescue sessions, and toolkits) between groups over time.

## Abbreviations

BHC: behavioral health coaching
CBT: cognitive behavioral therapy
EAP: employee assistance program

## Data Availability

The data analyzed in this study are not publicly available because of the restricted permissions and confidentiality shared between Intellect and its commercial partners. The data will only be available upon reasonable request made to the corresponding author.
