# Supplementary material for: Mobile Behavioral Health Coaching as a Preventive Intervention for Occupational Public Health: Retrospective Longitudinal Study
Source: JMIR Form Res. 2023 Oct 20;7:e45678. doi: 10.2196/45678 (PMC10625093; doi:10.2196/45678)
Supplement: Multimedia Appendix 1 [file formative_v7i1e45678_app1.docx]

**Appendix A**

| Table S1: Monthly Average Number of Learning Paths Completed between Groups | | | |
| --- | --- | --- | --- |
| Time Period by Month | Coaching | Control | *P-value* |
|  | *M (SD)* | |  |
| 1st Month | .820 (.755) | .720 (.714) | .03 |
| 2nd Month | .230 (.634) | .130 (.428) | .25 |
| 3rd Month | .180 (.556) | .150 (.484) | .46 |
| 4th Month | .170 (.632) | .200 (.545) | .65 |
| 5th Month | .150 (.535) | .160 (.535) | .94 |
| 6th Month | .180 (.573) | .130 (.549) | .55 |
| 7th Month | .190 (.593) | .210 (.682) | .85 |
| 8th Month | .210 (.609) | .210 (.740) | .99 |
| 9th Month | .240 (.665) | .330 (.913) | .60 |
| 10th Month | .220 (.702) | .410 (1.06) | .40 |
| 11th Month | .270 (.758) | .310 (1.01) | .87 |
| 12th Month | .280 (.751) | .080 (.277) | .36 |

| Table S2: Monthly Average Number of Journaling Sessions Completed between Groups | | | |
| --- | --- | --- | --- |
| Time Period by Month | Coaching | Control | *P-value* |
|  | *M (SD)* | |  |
| 1st Month | .880 (2.04) | .590 (1.73) | .01 |
| 2nd Month | .930 (2.80) | .650 (1.90) | .06 |
| 3rd Month | .910 (2.84) | .750 (1.95) | .41 |
| 4th Month | .400 (1.67) | .490 (1.69) | .74 |
| 5th Month | 1.14 (3.33) | 1.15 (2.70) | .97 |
| 6th Month | 1.15 (3.16) | 1.08 (2.32) | .87 |
| 7th Month | 1.65 (3.91) | 1.60 (2.85) | .94 |
| 8th Month | 1.46 (3.62) | 1.27 (2.60) | .79 |
| 9th Month | 2.82 (11.8) | 1.52 (2.70) | .62 |
| 10th Month | 1.90 (4.31) | 1.94 (2.86) | .97 |
| 11th Month | 2.16 (4.20) | 1.94 (3.04) | .85 |
| 12th Month | 3.30 (9.30) | 1.08 (2.75) | .40 |

| Table S3: Monthly Average Number of Rescue Sessions Completed between Groups | | | |
| --- | --- | --- | --- |
| Time Period by Month | Coaching | Control | *P-value* |
|  | *M (SD)* | |  |
| 1st Month | 1.08 (2.63) | .580 (1.40) | <.001 |
| 2nd Month | 1.07 (2.21) | .680 (1.50) | .06 |
| 3rd Month | .970 (2.37) | .930 (2.18) | .83 |
| 4th Month | .400 (1.67) | .490 (1.69) | .15 |
| 5th Month | 1.31 (2.92) | 1.68 (3.58) | .35 |
| 6th Month | 1.24 (2.55) | 1.17 (2.04) | .85 |
| 7th Month | 1.62 (3.50) | 1.95 (2.42) | .57 |
| 8th Month | 1.66 (2.89) | 2.03 (2.72) | .52 |
| 9th Month | 1.99 (3.89) | 1.90 (2.57) | .93 |
| 10th Month | 2.00 (3.41) | 1.88 (2.85) | .90 |
| 11th Month | 2.23 (2.88) | 1.88 (2.90) | .68 |
| 12th Month | 2.23 (3.10) | 1.46 (1.40) | .40 |

| Table S4: Monthly Average Number of Toolkit Sessions Completed between Groups | | | |
| --- | --- | --- | --- |
| Time Period by Month | Coaching | Control | *P-value* |
|  | *M (SD)* | |  |
| 1st Month | .960 (4.08) | 1.19 (10.4) | .64 |
| 2nd Month | 1.25 (4.83) | 1.21 (10.2) | .93 |
| 3rd Month | 1.17 (4.90) | 1.68 (13.3) | .48 |
| 4th Month | 1.49 (5.30) | 2.71 (17.7) | .28 |
| 5th Month | 1.92 (6.66) | 3.86 (23.2) | .27 |
| 6th Month | 2.20 (7.17) | 4.52 (28.0) | .34 |
| 7th Month | 2.77 (8.21) | 7.26 (34.6) | .20 |
| 8th Month | 2.66 (8.15) | 8.52 (38.9) | .18 |
| 9th Month | 2.72 (7.60) | 12.5 (48.7) | .10 |
| 10th Month | 2.60 (6.66) | 16.1 (53.9) | .06 |
| 11th Month | 2.27 (6.42) | 16.8 (55.6) | .09 |
| 12th Month | 1.33 (2.97) | 24.1 (78.3) | .06 |
